# Virulence attenuation in ST11-K64 *Klebsiella pneumoniae* explains its divergent clinical manifestation from ST23-K1

**DOI:** 10.1080/21505594.2026.2690744

**Published:** 2026-06-26

**Authors:** Shujing Li, Ying Zhang, Qian Zeng, Lilan Zheng, Jing Yan, Yong Wang, Yun Liu, Lianhua Yu, Yi Li, Meng Li, Jun Feng, Xiaofei Jiang

**Affiliations:** a Department of Laboratory Medicine, Huashan Hospital, Fudan University, Shanghai, China; b Department of Laboratory Medicine, Obstetrics & Gynecology Hospital of Fudan University, Shanghai Key Lab of Reproduction and Development, Shanghai Key Lab of Female Reproductive Endocrine Related Diseases, Shanghai, China; c Department of Laboratory Medicine, Jinshan Hospital of Fudan University, Shanghai, China; d Department of Clinical Laboratory, Shandong Provincial Hospital Affiliated to Shandong First Medical University, Jinan, Shandong, China; e Department of Laboratory Medicine, Taizhou Municipal Hospital, Taizhou, Zhejiang, China; f Department of Laboratory Medicine, Henan Provincial People’s Hospital, Zhengzhou, Henan, China; g Department of Clinical Laboratory, The First Affiliated Hospital of Guangxi Medical University, Nanning, Guangxi, China; h Department of Pathogen Detection, Shanghai Municipal Center for Disease Control and Prevention, Shanghai, China

**Keywords:** *Klebsiella pneumoniae*, carbapenem-resistant, hypervirulent, pathogenicity, intestinal translocation, liver abscess

## Abstract

The clinical threat posed by *Klebsiella pneumoniae* is dual-faceted, encompassing both hypervirulence and carbapenem resistance. The emergence of hypervirulent carbapenem-resistant *K. pneumoniae* (hv-CRKP) merges these threats, with the ST11-K64 clone being a dominant and concerning lineage. However, its clinical presentation diverges from the classic hypervirulent *Klebsiella pneumoniae* (hvKp); hv-CRKP is isolated from respiratory sites but is notably absent from pyogenic liver abscesses. This distinct clinical niche prompted us to investigate the underlying pathogenicity differences. Among 847 clinical *Klebsiella pneumoniae* isolates, 157 (18.5%) were identified as hv-CRKP. From this hv-CRKP collection, we selected five representative ST11-K64 isolates for downstream phenotypic and mechanistic analyses. Despite its prevalence and multidrug resistance, the ST11-K64 clone exhibited significantly attenuated lethality in mouse models compared to ST23-K1. Crucially, in a murine intestinal colonization model that mimics natural infection, only ST23-K1 successfully colonized the gut, caused bacteremia, and formed liver abscesses. In contrast, ST11-K64 strains showed impaired intestinal colonization and failed to translocate to the liver. Phenotypic profiling showed reduced capsule viscosity and siderophore production in ST11-K64 relative to ST23-K1, accompanied by diminished macrophage and Kupffer cell-associated fitness. RT-qPCR identified higher expression of *rmpA*, *rmpA2*, *iroB,* and *iucA* in ST23-K1, and isogenic deletion and complementation of *rmpA2* or *iucA* in the ST23-K1 background supported their contribution to capsule/siderophore-associated phenotypes and intracellular survival in RAW264.7 macrophages. Together, these results indicate that while ST11-K64 hv-CRKP represents a serious antimicrobial-resistance threat, its invasive pathogenicity is route- and context-dependent and does not fully recapitulate the classic entero-hepatic hypervirulence of ST23-K1.

## Introduction

*Klebsiella pneumoniae* (Kp) is a major cause of healthcare-associated and community-acquired infections, with its clinical severity amplified by two concerning evolutionary paths: hypervirulent *Klebsiella pneumoniae* (hvKp) that trigger life-threatening invasive syndromes like pyogenic liver abscesses in healthy hosts [1,2], and carbapenem-resistant *Klebsiella pneumoniae* (CRKP) carrying carbapenemases that drive untreatable outbreaks in immunocompromised hosts [3]. The convergence of these lineages into hypervirulent carbapenem-resistant *K. pneumoniae* (hv-CRKP) has spurred alarm about an emergent “perfect storm” pathogen [4–6], leading to a molecular-centric definition based on detecting virulence genes alongside carbapenemase genes [7]. This approach classifies ST11-K64 strains carrying both resistance and virulence genes as hv-CRKP. However, despite their genetic profile, ST11-K64 isolates are infrequently linked to invasive diseases like liver abscesses, which remain dominated by ST23-K1 strains [8,9]. Although attenuated virulence in hv-CRKP has been noted [10,11], the specific stage of failure to cause invasive disease and its underlying mechanisms are unknown.

To bridge this gap, we selected 5 representative hv-CRKP strains from 157 hv-CRKP isolates among 847 clinical samples. An integrated strategy employing Galleria mellonella and murine sepsis models, murine intestinal colonization-liver translocation models (mimicking the natural human infection route [12]), quantitative tracking of bacterial burden across sequential host barriers, cellular assays, and virulence factor analyses was used to delineate the precise stage and mechanism of their attenuated pathogenicity in the gut–liver translocation pathway. Our study clarifies the differences between ST23-K1 hvKp and ST11-K64 hv-CRKP in terms of pathogenicity in liver abscess. We found that although the ST11-K64 hv-CRKP carries more virulence genes than classical CRKP, it shows reduced ability to colonize the gut and survive in the liver compared to the ST23 strain. These results suggest that ST11-K64 hv-CRKP may not represent a typical hypervirulent clone, which encourages a more nuanced understanding of bacterial pathogenicity and supports efforts to prevent the emergence of hypervirulent and carbapenem-resistant strains.

## Methods and materials

### Whole-genome sequencing and analysis

A total of 847 clinical *Klebsiella pneumoniae* isolates were collected from four hospitals in China during the study period. To avoid duplicate sampling, only one isolate per patient was included in the analysis.

Genomic DNA was extracted and purified from all 847 clinical *Klebsiella pneumoniae* isolates using the TIANamp Bacterial DNA Kit (TIANGEN Biotech; DP302). The resulting DNA was then sequenced using the HiSeq X Ten PE150 platform (Illumina, USA). Quality-controlled reads were obtained using CLC Genomics Workbench 12.0 (QIAGEN, Aarhus, Denmark), where raw data were filtered by removing sequences with >10% ambiguous bases or shorter than 30 bp. De novo assembly was then performed with default settings.

Sequence types (STs), ARGs, virulence-associated genes, resistance score, virulence score, capsule serotype (K), and O antigen (LPS) serotype predictions were identified using Kleborate 3.0. Capsule serotype (K) and O-antigen serotype predictions were based on Kleborate outputs and are reported as genotype-based inferences. Predictions flagged by Kleborate as untypeable were recorded as “undetermined” and were not used for strong lineage-level claims regarding capsule type prevalence. A core single-nucleotide polymorphisms (SNP)-based tree was established by PGCGAP 1.0.35.

### PCR confirmation of MLST and capsule type

To confirm the genotypes of the representative isolates selected for downstream experiments, sequence type (ST) and capsule type were verified by PCR and Sanger sequencing. For MLST, internal fragments of the seven housekeeping genes of the *Klebsiella pneumoniae* Pasteur scheme (*gapA, infB, mdh, pgi, phoE, rpoB, tonB*) were amplified. For capsule typing, the *wzi* gene fragment was amplified. PCR products were purified and subjected to Sanger sequencing, and the resulting sequences were compared against the Institut Pasteur MLST database to assign allele numbers and determine the corresponding ST; wzi sequences were similarly queried against the Pasteur database to support capsule type assignment.

### Mouse lethality assay

All procedures were performed in accordance with laboratory animal care and use guidelines of the Chinese Association for Laboratory Animal Sciences. Approval was obtained from the Huashan Hospital Animal Care and Use Committee (2025-HSYY-053). Six- to eight-week-old female BALB/c mice (*n* = 8 per group) were purchased from SPF (Beijing) Biotechnology Co., Ltd. and maintained under standard conditions. Bacterial strains were grown to mid-log phase, harvested, washed, and resuspended in 1 ml PBS (Servicebio, G4207-500 ML). For the infection procedure, mice were anesthetized by intraperitoneal injection of tribromoethanol (2.5%, 0.2 ml/10 g). Mice were then intraperitoneally injected with 100 μL of bacterial suspension (5 × 10^7^ CFU [13]). Survival was monitored every 12 h for 72 h, and moribund mice were euthanized by CO_2_ inhalation followed by cervical dislocation to ensure death.

### Galleria mellonella lethality assay

Galleria mellonella larvae (approximately 25–30 mg) were acclimatized at 25°C for 24 h. Groups of larvae (*n* = 10) were injected in the last abdominal segment with 10 μL of bacterial suspension (5 × 10^6^ CFU/larva [13]) using a microsyringe. Inoculated larvae were incubated at 37°C in Petri dishes, and survival was monitored every 12 h for 72 h. Death was defined as absence of movement upon gentle prodding.

### Mouse oral gavage assay for liver abscess induction

All animal procedures were approved by the Laboratory Animal Welfare and Ethics Committee of the Fudan University Experimental Animal Center (approval no. 2025-HSYY-053). Female BALB/c mice (6–7 weeks old, *n* = 5 per group) were used in two independent oral gavage experiments. Bacteria were grown to mid-log phase (OD_6__00_ ≈ 0.5–0.6), harvested, washed with sterile PBS, and resuspended at the required density.

For the initial liver abscess induction experiment, mice were acclimatized for 1 week and then gavaged daily with 200 µL of bacterial suspension (2 × 10^8^ CFU/mouse [14,15]) for 7 consecutive days, with ad libitum access to food and water. The strains tested included NTUH-K2044, D341, D345, D386, D434, D441, HS11286. On day 8, mice were deeply anesthetized with tribromoethanol (2.5%, 0.2 mL/10 g body weight, i.p.) and euthanized by cervical dislocation. The small intestine, large intestine, feces, blood, and liver were collected. Liver tissue was fixed in formalin for H&E staining and histopathological analysis, and the remaining tissues were homogenized for bacterial enumeration (CFU/g or CFU/mL).

To shorten the experimental timeline and improve the success rate of infection, a modified protocol was subsequently developed for virulence assessment of isogenic mutants. NTUH-K2044, its isogenic mutants ΔiucA and ΔrmpA2, and the corresponding complemented strains (ΔiucA:iucA and ΔrmpA2:rmpA2) were used. After 5 d of acclimation, mice received a higher inoculum of 1–2 × 10^9^ CFU/mouse (200 µL) by oral gavage once daily for 5 d. A strict fasting/water-deprivation regimen was applied: before the first gavage, mice were fasted for 16 h (with free access to water) and then deprived of water for 4 h; after the first gavage, both food and water were withheld for 4 h. For gavages on days 2–5, the same 4‑h food and water deprivation was imposed before and after each gavage. On day 6, mice were anesthetized and euthanized as above, and the small intestine, large intestine, feces, blood, liver, spleen, and lungs were harvested for CFU enumeration.

### H&E staining and histopathological analysis

Liver tissues were fixed for ≥24 h, rinsed, dehydrated through graded ethanol, cleared, and paraffin-embedded. Paraffin blocks were sectioned at a thickness of 4 μm; sections were floated on a 42°C water bath and baked at 60°C for 30 min–2 h. For H&E staining, sections were deparaffinized and rehydrated, stained with Harris hematoxylin for 4 min, differentiated with 0.8% acid alcohol, blued, counterstained with eosin for 20 s, dehydrated, cleared, and mounted. Stained slides were scanned using a NanoZoomer S360 digital slide scanner and viewed with NDP.view v2.9.22; images shown in the manuscript were captured at 20× magnification, with a 10× ocular lens, the total magnification was 200× .

### Caco-2/HepG2 adhesion and invasion assays

The experimental procedures employed in this work were based on a previously reported protocol [16], with slight adjustments. Caco-2/HepG2 cells (QuiCell Biotechnology, Shanghai) were maintained in DMEM with 20% FBS/10% FBS at 37°C and 5% CO_2_. Cells were seeded in 24-well plates at 1 × 10^5^ cells/well. Bacteria were grown to mid-log phase (OD_6__00_ ≈ 0.5–0.6), washed, and resuspended in antibiotic-free DMEM. For adhesion assays, cells were infected at an MOI of 20:1, centrifuged (500 × g, 5 min), and incubated for 30 min. After washing five times with PBS, cells were lysed with 200 ul 0.2% Triton X-100 (Sangon Biotech (Shanghai) Co., Ltd; V900502-500 ML), and lysates were plated to quantify adherent bacteria. For invasion assays, following a 2 h infection, extracellular bacteria were eliminated by incubation with 500 μg/mL tigecycline for 1.5 h before washing, lysis, and plating.

### RAW264.7 intracellular survival assay

RAW264.7 macrophages (QuiCell Biotechnology) were cultured in DMEM with 10% FBS. Cells were seeded overnight in 24-well plates at 5 × 10^5^ cells/well. Bacteria were grown to mid-log phase, washed, and resuspended in antibiotic-free DMEM. Macrophages were infected at an MOI of 20:1, synchronized by centrifugation (500 × g, 5 min), and incubated for 2 h. Extracellular bacteria were removed by washing and exposed to 500 μg/mL tigecycline (Sangon Biotech (Shanghai) Co., Ltd; A427324-0005) for 1 h, followed by replacement with medium containing 10 μg/mL tigecycline. At 0, 8, 16, 24, and 48 h post-infection, cells were lysed with 0.2% Triton X-100, and intracellular bacteria were quantified by plating. Survival percentage was calculated by normalizing CFU at each time point to the mean 0 h value across all strains.

### Immortalized Kupffer cells (ImKcs) intracellular survival assay

Immortalized Kupffer cells have been demonstrated to function as effective substitutes for primary Kupffer cells in experimental settings and are widely available commercially [17,18]. Considering practical factors such as cellular stability and cost-effectiveness, we employed ImKCs for the current study. ImKCs (Ethephon Biotechnology, Shanghai) were maintained in DMEM with 10% FBS at 37°C and 5% CO_2_. Cells were seeded in 24-well plates at 5 × 10^5^ cells/well and incubated overnight. Bacteria were grown to mid-log phase (OD_6__00_ ≈ 0.5–0.6), washed, and resuspended in antibiotic-free DMEM. ImKCs were infected at an MOI of 20:1, centrifuged (500 × g, 5 min), and incubated for 2 h to allow phagocytosis. After PBS washing, extracellular bacteria were killed by treatment with 500 μg/mL tigecycline for 1 h, followed by replacement with a medium containing 10 μg/mL tigecycline. At 0, 2, 4, 16, 20, and 24 h post-infection, cells were lysed with 0.2% Triton X-100, and intracellular bacteria were enumerated by plating. Intracellular survival percentage was calculated as the normalized CFU at each time point relative to the normalized 0 h value for each strain.

### Serum resistance assay

Normal human serum was obtained from healthy donors, and the serum killing assay was performed as previously described [19]. Bacterial strains were grown to mid-log phase, washed, and resuspended to OD_6__00_ = 0.3. Then, 250 μL of bacterial suspension was mixed with 750 μL of serum and incubated at 37°C with shaking (20 rpm) for 2 h. Aliquots were collected at 0 and 2 h, serially diluted in PBS, and plated on LB agar. CFUs were enumerated after overnight incubation at 37°C. Survival was calculated as CFU at 2 h relative to CFU at 0 h.

### Capsule viscosity semi-quantitative assay

Capsule mucoviscosity (sedimentation) assay was performed as previously described with minor modifications [20]. Bacterial strains were cultured overnight in LB at 37°C with shaking (200 rpm). For each strain, 1 mL culture was centrifuged (12,000 rpm, 15 min) to pellet cells and the supernatant was discarded. Pellets were resuspended in 1 mL PBS and centrifuged (1,000 × g, 5 min). The supernatant OD_6__00_ was measured, and the OD_6__00_ of the original overnight culture was measured in parallel. Relative capsule viscosity was calculated as supernatant OD_6__00_/culture OD_6__00_.

### Capsule extraction and uronic acid quantification

Capsular polysaccharide (CPS) quantification was performed as previously described [4,20]. Capsular polysaccharide was extracted from 500 μL of bacterial culture by adding 100 μL of 1% Zwittergent 3–14 (in 100 mM citrate buffer) (SANTACRUZ; sc-281193), incubating at 50°C for 20 min, and centrifuging at 13,000 × g for 5 min. The supernatant (300 μL) was mixed with 1.2 mL 80% ethanol (Sangon Biotech (Shanghai) Co., Ltd ;A375262-0001), incubated on ice for 20 min, and centrifuged again. The CPS pellet was air-dried, resuspended in 200 μL ddH_2_O, mixed with 1.2 mL sodium borate solution (12.5 mM in H_2_SO_4_) (bioss; D10122-250 g), and hydrolyzed at 100°C for 5 min. After cooling on ice, uronic acid was quantified by adding 20 μL meta-hydroxydiphenyl solution (0.15% in 0.5% NaOH) (yuanye; S30798-5 g), incubating for 5 min, and measuring absorbance at 520 nm [49]. Concentrations were determined using a glucuronic acid standard curve. Data are presented as mean ± SD from three independent experiments.

### Siderophore qualitative assay

The Chrome Azurol S (CAS) detection solution was prepared by separately dissolving Chrome Azurol S (60.5 mg in 50 mL H_2_O) (Yuanye; S19255-500 g), FeCl_3_·6 H_2_O (1 mM in 10 mM HCl) (Perfemiker; PA54241-100 g), and HDTMA (72.9 mg in 40 mL H_2_O) (Lablead; 0833-50 g). The FeCl_3_ and CAS solutions were combined and slowly added to the HDTMA solution with stirring. The resulting CAS – HDTMA – Fe^3 +^ complex was autoclaved (121°C, 15 min) and stored in the dark. For CAS double-layer plates, King’s B agar (dissolved in 700 mL H_2_O, autoclaved) was cooled to 50–60°C, mixed with 200 mL 10× PIPES buffer (pH 6.8) and 100 mL CAS solution, and 15 mL were poured as the bottom layer. After solidification, 5 mL of sterile LB Agar was overlaid. A 1 μL aliquot of logarithmic-phase culture was spotted onto the plates and incubated at 37°C for 36–48 h. Siderophore production was indicated by an orange-yellow halo around colonies.

### Siderophore relative quantification assay

Inoculate a single fresh colony into MKB medium and culture at 37°C with shaking for 20–24 h. Centrifuge 1 mL of culture at 12,000 rpm for 10 min to obtain cell-free supernatant. Prepare CAS detection solution containing 1.2 mM CTAB, 2 mM CAS, and 1 mM FeCl_3_·6 H_2_O. Add 100 μL of supernatant to triplicate wells of a 96-well plate, followed by 100 μL CAS solution. Incubate in the dark for 30 min, then measure absorbance at 630 nm (As). Use sterile MKB medium as a reference control (Ar). Calculate siderophore units (Su) using [4,21]: Su (%) = [(Ar−As)/Ar]×100.

### Quantitative RT-PCR

Total RNA was isolated from bacteria harvested during the logarithmic growth phase and reverse-transcribed into cDNA using the PrimeScript™ RT Reagent Kit. Quantitative real-time PCR was carried out on a LightCycler® System. Relative transcript levels were normalized to rpoB and calculated using the 2^−ΔΔCt method based on Ct values. Three independent biological replicates were analyzed for each strain.

### Construction of gene knockout strains and plasmids

The gene knockout strains K2044ΔwcaJ and K2044ΔrmpA were constructed using the λ-Red homologous recombination system, as previously reported [22].

Plasmids pACYC-iucA and pACYC-rmpA2 were constructed using the NEBuilder HiFi DNA Assembly Cloning Kit (NEB, M0494), following the manufacturer’s recommended protocol. Additionally, the pACYC184 plasmid was electrotransferred into NTUH-K2044 as a negative control.

### Statistical analysis

All experiments were conducted with three independent biological replicates unless otherwise specified. Data were visualized and statistically analyzed using GraphPad Prism software (v9.0.0). Parametric assumptions, including normality (Shapiro–Wilk test) and homogeneity of variance (Brown–Forsythe test), were verified prior to statistical comparisons. For two-group comparisons, a two-tailed, unpaired Student’s t-test was employed. Datasets involving multiple groups and variables were analyzed by two-way analysis of variance (ANOVA) followed by Tukey’s post hoc test. A *p*-value of less than 0.05 was considered statistically significant.

### Ethics statement

Animal experiments were approved by the Laboratory Animal Welfare and Ethics Committee of the Fudan University Experimental Animal Center (approval no. 2025-HSYY-053) and were conducted in accordance with institutional guidelines for the care and use of laboratory animals.

The use of clinical bacterial isolates and human serum samples in this study was reviewed and approved by the Ethics Committee of Huashan Hospital, Fudan University, China (Approval No. 2021–484). The clinical isolates were collected from routine microbiological diagnostics at participating hospitals and were anonymized before analysis. No identifiable patient information was collected or used in this study. The requirement for informed consent from patients was waived by the Ethics Committee due to the retrospective use of anonymized bacterial isolates. Human serum samples were obtained from healthy volunteers with written informed consent. All procedures involving human-derived materials were conducted in accordance with the Declaration of Helsinki.

## Results

### Multicenter epidemiological survey of Klebsiella pneumoniae and characterization of hv-CRKP isolates

**Figure 1.**
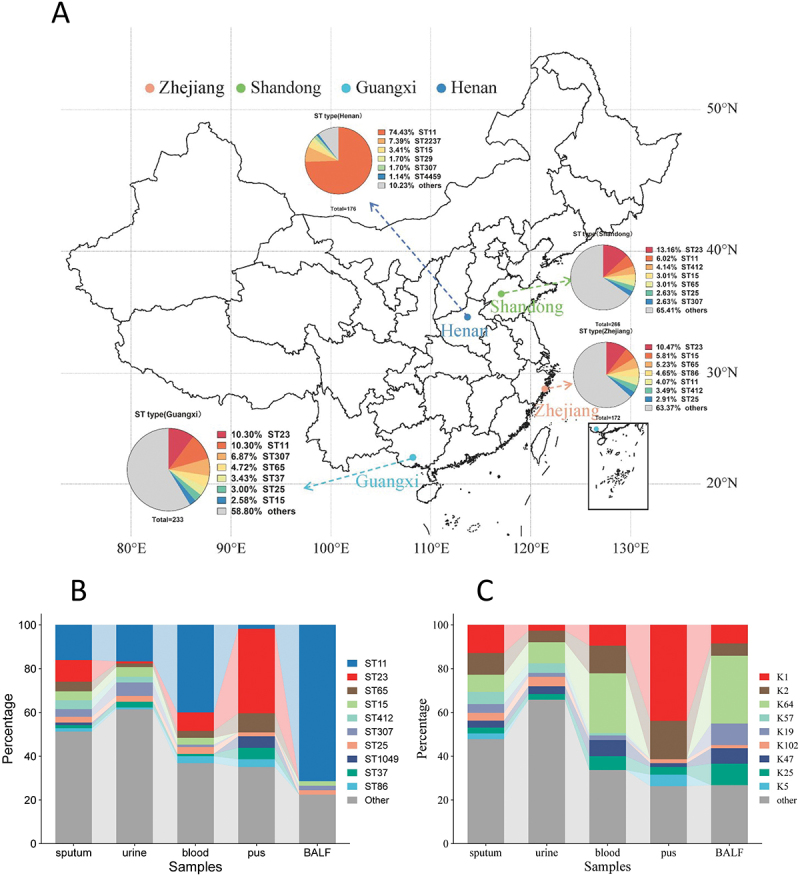
Molecular epidemiology of *Klebsiella pneumoniae* isolates from four Chinese hospitals.

Molecular epidemiological analysis of 847 clinical *Klebsiella pneumoniae* isolates from our cohort revealed significant geographical heterogeneity in sequence type distribution, with ST11 and ST23 showing distinct regional dominance patterns (Figure 1(A)). Further stratification by specimen source demonstrated clear niche specificity: while ST11 and K64 consistently dominated most specimen types, ST23 and K1 serotypes exhibited a striking predominance in pus samples, suggesting their specific adaptation to pyogenic infections (Figure 1(B,C)).

(A) Geographic distribution of predominant sequence types (STs). Pie charts depict the relative abundance of the top seven most prevalent STs among *K. pneumoniae* clinical isolates collected from hospitals in Shandong, Henan, Zhejiang, and Guangxi provinces. STs not ranked within the top seven per region are grouped as “Others.” (B) Distribution of STs across sample types. Bar chart showing the percentage distribution of the 10 most common STs and other STs among all K. pneumoniae isolates. (C) Distribution of capsular types (K types) across sample types. Bar chart showing the percentage distribution of the nine most common K types and other K types among all K. pneumoniae isolates.

**Figure 2.**
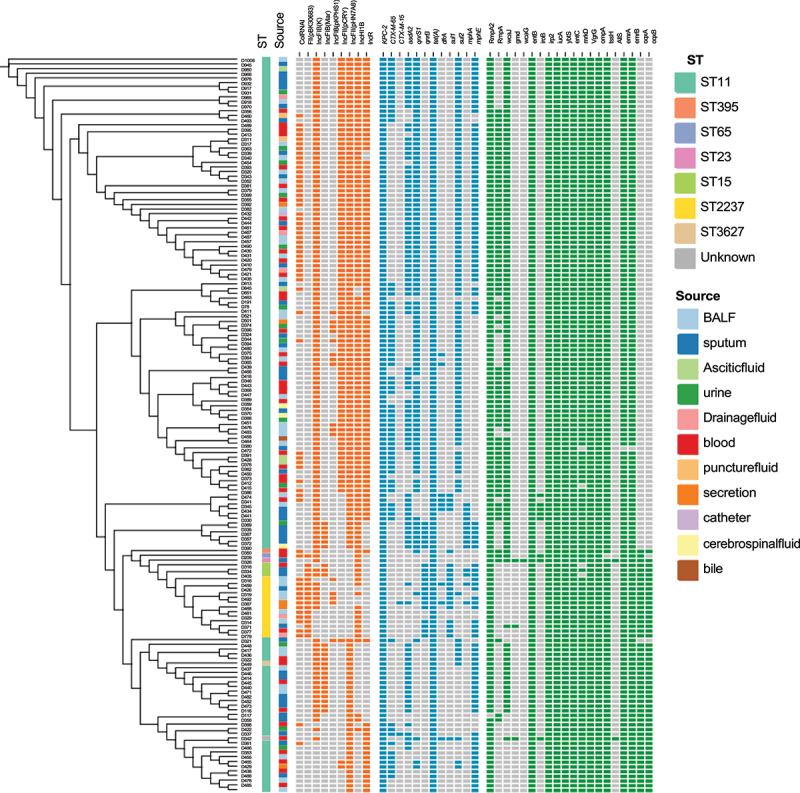
Core SNP phylogeny and Kleborate-derived feature heatmap of hv-CRKP isolates.

Among 847 clinical *Klebsiella pneumoniae* we collected, 157 isolates (18.5%, 95% CI: 16.1–21.3) were identified as hv-CRKP based on co-carriage of key virulence genes (*rmpA/rmpA2* and *iucA/iroB*) and *blaKPC* (Figure S1A, Figure S2A, Figure 2). These dual-risk isolates were primarily recovered from respiratory specimens and showed strong clonal predominance: Among the 157 hv-CRKP isolates, 93 were ST11-K64, accounting for 59.2% (95% CI: 51.4–66.6) (Figure S1B, Figure S2B). Based on Kleborate-derived virulence gene calls, *rmpA* was detected in 80/157 isolates (50.96%), whereas 77/157 isolates (49.04%) lacked *rmpA* (Figure 2).

A core SNP-based phylogenetic tree was constructed using PGCGAP v1.0.35. Adjacent annotations indicate each isolate’s sequence type (ST) and specimen source. The heatmap summarizes ARGs, virulence-associated genes, and related genotypic features identified by Kleborate v3.0, where colored tiles denote presence and gray tiles denote absence. Capsule (K) and O-antigen (LPS) serotypes, when shown, are genotype-based predictions from Kleborate.

Five representative ST11 isolates (D341, D345, D386, D434, and D441) (Figure S3), all recovered from respiratory specimens, were selected for subsequent experiments because genomic screening of our hv-CRKP collection identified these strains as concurrently carrying the key virulence markers *rmpA, rmpA2, iucA, and iroB*, together with *blaKPC-2*. To further reconfirm the genotypes of the selected isolates, we performed PCR-based typing followed by Sanger sequencing to verify both the ST (Pasteur MLST housekeeping genes) and the capsule type (*wzi*-based K typing). Antimicrobial susceptibility testing further showed that all five ST11 isolates were resistant to carbapenems and extended-spectrum β-lactams (Table S1).

### Hypervirulent carbapenem-resistant K. pneumoniae exhibits attenuated lethality in murine model and is unable to establish liver abscess via oral inoculation

**Figure 3.**
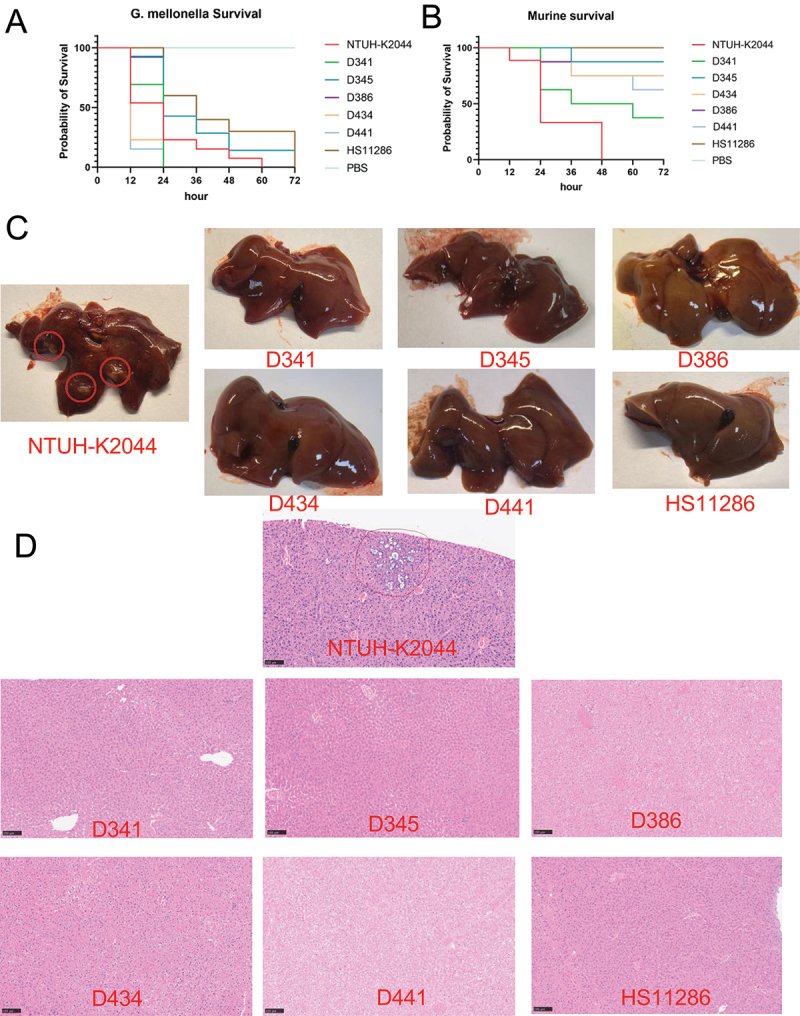
Virulence assessment and hepatic pathogenicity of *K. pneumoniae* strains in infection models.

Virulence was assessed using Galleria mellonella and murine lethality assays. Using a Galleria mellonella model (5 × 10^6 CFU/larva) and a murine intraperitoneal challenge model (5 × 10^7 CFU/mouse), we assessed the lethality of representative ST11-K64 hv-CRKP isolates relative to NTUH-K2044. The larval model did not clearly differentiate strains (Figure 3(A)), whereas the mouse intraperitoneal model showed reduced lethality for ST11-K64 hv-CRKP isolates, only strain D341 approached the high lethality of NTUH-K2044 (Figure 3(B)). To specifically evaluate their capacity to cause pyogenic liver abscesses, an oral inoculation model mimicking the entero-hematogenous route was employed. Macroscopic and histopathological examinations showed that characteristic liver abscesses were induced exclusively by NTUH-K2044, while none of the hv-CRKP strains produced such pathological changes (Figure 3(C,D)). These results indicate that although hv-CRKP strains exhibit enhanced lethality compared to classical CRKP, they lack the complete pathogenic capability of classical hypervirulent K. pneumoniae to establish invasive liver infection via the intestinal tract.

(A) Kaplan–Meier survival curves of G. mellonella larvae injected with 5 × 10^6 CFU/larva. (B) Kaplan–Meier survival curves of BALB/c mice intraperitoneally injected with 5 × 10^7 CFU per mouse. (C) Gross morphology of murine livers, showing abscesses only in the NTUH-K2044-infected group. (D) Representative H&E-stained liver sections, revealing significant necrotic lesions exclusively in mice infected with NTUH-K2044 (areas outlined in red). All images were acquired at 400× total magnification; scale bar, 100 μm (lower left).

**Figure 4.**
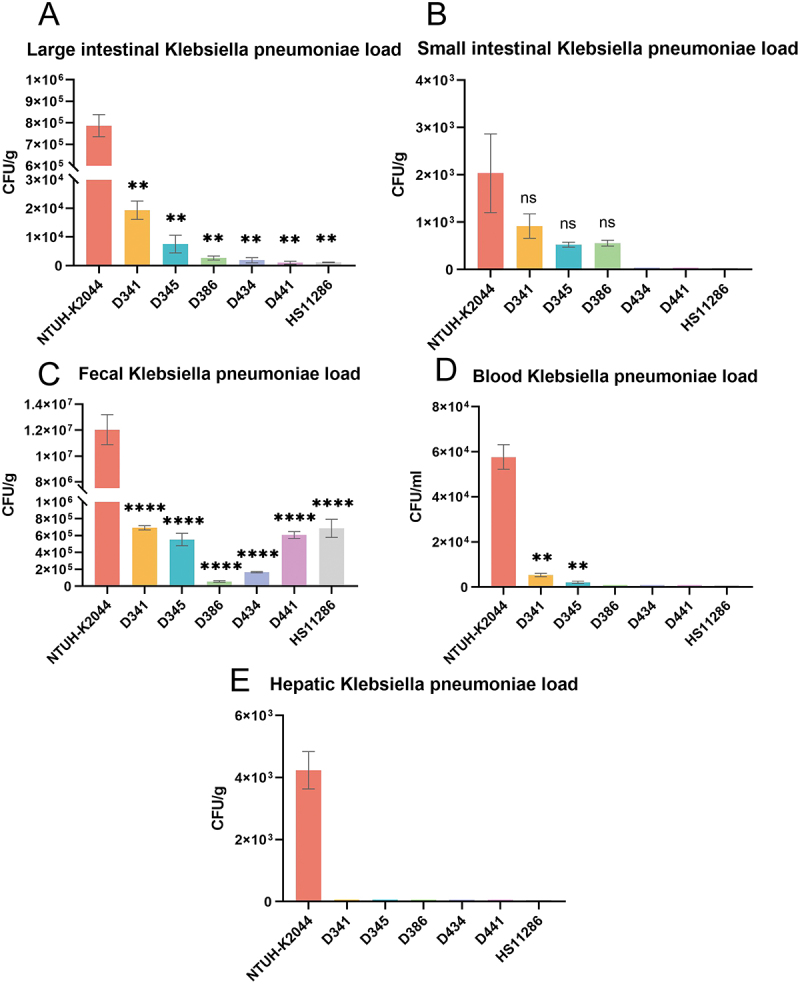
Bacterial loads of *Klebsiella pneumoniae* strains in various tissues and body fluids after continuous oral infection.

To identify the divergence point in the entero-hematogenous-hepatic pathway between hvKp and hv-CRKP, bacterial loads in key tissues were quantified after 7 d of oral gavage in mice. NTUH-K2044 demonstrated superior colonization, with mean fecal burdens of 1.20 × 10^7 ± 1.16 × 10^6 CFU/g and large-intestine loads of 7.87 × 10^5 ± 5.13 × 10^4 CFU/g, whereas the five ST11-K64 hv-CRKP isolates showed markedly lower levels overall (feces: 4.15 × 10^5 ± 2.65 × 10^5 CFU/g; large intestine: 6.49 × 10^3 ± 7.26 × 10^3 CFU/g, pooled across five isolates) (Figure 4(A,C)). Bacterial levels in the small intestine remained low across all strains (Figure 4(B)). Consistent with systemic dissemination, NTUH-K2044 alone achieved high-level bacteremia (5.77 × 10^4 ± 5.51 × 10^3 CFU/mL), while only D341 and D345 were detectable in blood at much lower levels (5.37 × 10^3 ± 7.02 × 10^2 and 2.10 × 10^3 ± 5.00 × 10^2 CFU/mL, respectively) (Figure 4(D)). Importantly, no bacteria were recovered from the liver for any hv-CRKP isolate, whereas NTUH-K2044 established a detectable liver infection (4.23 × 10^3 ± 6.03 × 10^2 CFU/g) (Figure 4(E)). This indicates that hv-CRKP strains are impaired in intestinal colonization and subsequent liver establishment, failing to replicate the full pathogenicity of the hypervirulent prototype.

(A) Bacterial loads in the large intestine. (B) Bacterial loads in the small intestine. (C) Bacterial loads in fecal samples. (D) Bacterial loads in blood. (E) Bacterial loads in the liver. Data are presented as mean ± standard deviation (SD). Statistical significance was determined by one-way analysis of variance (ANOVA) with Tukey’s post hoc test. ns: not significant; *p* < 0.05 (*), *p* < 0.01 (**), *p* < 0.0001 (****).

### Compared to cellular adhesion and invasion, the capacity for immune evasion may be the pivotal capability enabling hvKp to successfully translocate from intestine to liver

To investigate the mechanistic basis for the enhanced colonization of NTUH-K2044 in the large intestine and liver, we first assessed its adhesive and invasive capacities using Caco-2 and HepG2 cell lines. Adhesion assays showed that NTUH-K2044 exhibited weaker binding to Caco-2 cells compared to the hv-CRKP isolates and HS11286 (Figure S4(A)), while its adhesion to HepG2 cells was comparable to that of hv-CRKP strains but lower than HS11286 (Figure S4(B)). Invasion assays revealed no significant differences among strains in Caco-2 cells (Figure S4(C)); however, in HepG2 cells, hv-CRKP strains displayed significantly greater invasiveness than both NTUH-K2044 and HS11286 (Figure S4(D)). These results indicate that the colonization advantage of NTUH-K2044 is unlikely attributable to superior epithelial adhesion or invasion.

**Figure 5.**
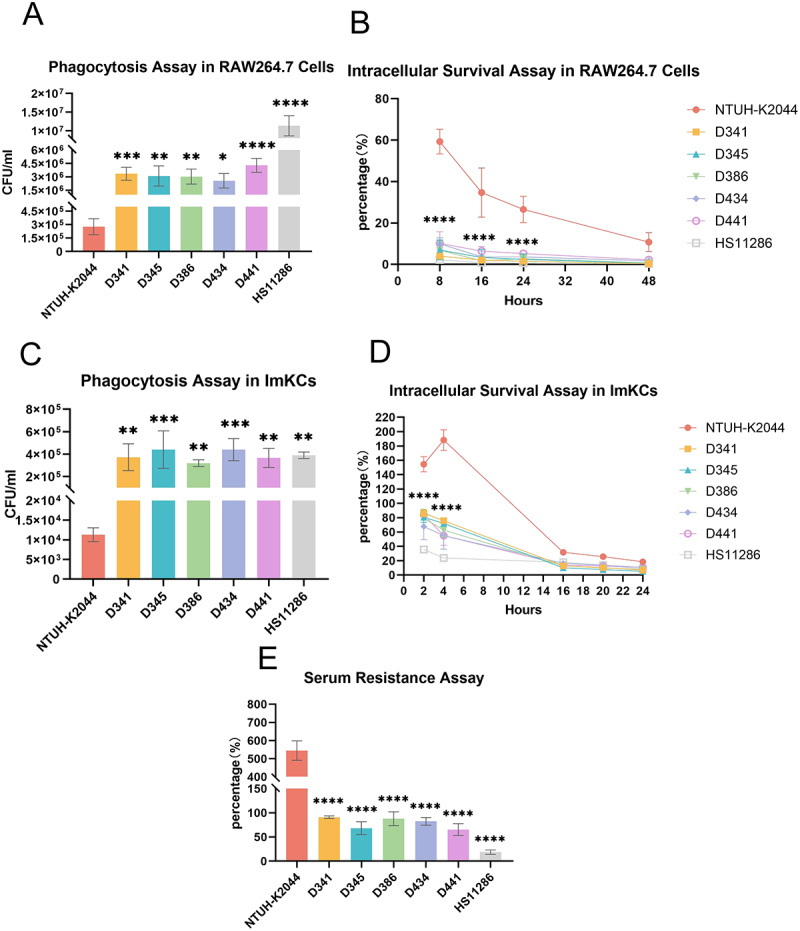
Immune evasion phenotypes of *K. pneumoniae* strains.

We therefore focused on immune evasion mechanisms. NTUH-K2044 demonstrated significantly stronger resistance to macrophage phagocytosis compared to hv-CRKP and HS11286 (Figure 5(A,C)); for example, in RAW264.7 cells, the recovered intracellular CFU after uptake was on the order of 10^5 for NTUH-K2044 (1.8–4.1 × 10^5 CFU/mL), but 10^6 for hv-CRKP (1.3–5.2 × 10^6 CFU/mL) and even higher for HS11286 (8.4–16 × 10^6 CFU/mL). Even upon internalization, intracellular survival of NTUH-K2044 was sustained longer (Figure 5(B)), with 53–65% remaining at 8 h, whereas hv-CRKP isolates generally declined to 3.5–16.6% by 8 h and to 0.97–8.5% by 16 h, consistent with enhanced resistance to intracellular killing. In immortalized Kupffer cells (ImKCs), NTUH-K2044 showed a transient proliferative trend within the first 4 h (Figure 5(D)), rising to 143–204% of the starting inoculum at 2–4 h and maintaining higher subsequent survival than other strains. Serum killing assays further indicated that NTUH-K2044 largely evaded complement-mediated killing, increasing to 485–587% relative to 0 h, whereas hv-CRKP strains showed intermediate serum resistance (56–99%) compared with HS11286 (~15–24%) (Figure 5(E)). Together, these findings suggest that the pronounced ability of NTUH-K2044 to resist both phagocytic and serum-mediated killing underlies its successful colonization and pathogenicity in intestinal and hepatic niches.

(A) Phagocytosis by RAW264.7 macrophages. NTUH-K2044 was significantly less phagocytosed than all other strains. (B) Intracellular survival in RAW264.7 cells over 48 h. NTUH-K2044 exhibited sustained survival, while other strains were cleared more rapidly. (C) Phagocytosis by immortalized Kupffer cells (ImKCs). NTUH-K2044 showed significantly lower uptake. (D) Intracellular survival in ImKCs over 24 h. NTUH-K2044 maintained a higher bacterial load, with an initial proliferative trend within 4 h. (E) Survival in normal human serum (mainly complement-mediated killing). Survival is expressed as CFU at 2 h relative to the 0 h inoculum. NTUH-K2044 showed the highest survival, whereas hv-CRKP isolates displayed intermediate survival compared with HS11286.

### Comparative analysis of capsular polysaccharide and siderophore production in hvKp, hv-CRKP, and CRKP

**Figure 6.**
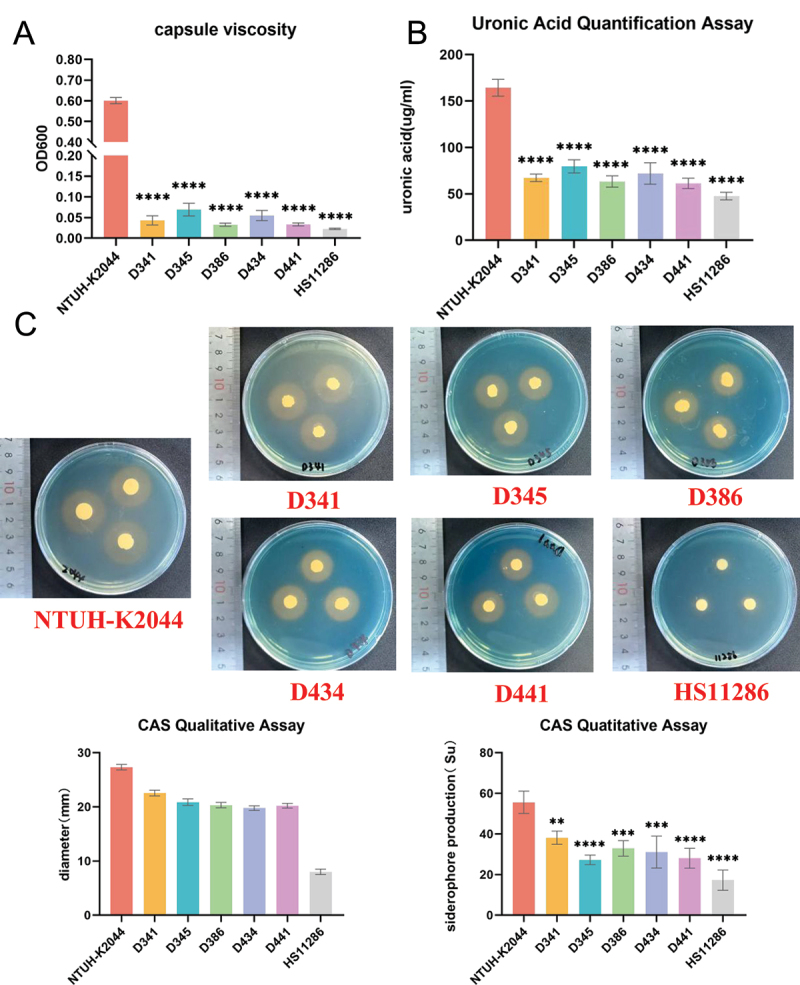
Characterization of capsule properties and siderophore production in different *Klebsiella pneumoniae* strains.

Having confirmed the enhanced immune resistance of hvKp, we further sought to elucidate differences in virulence factors between NTUH-K2044, hv-CRKP isolates, and HS11286. Capsular viscosity assays and quantitative uronic acid measurements revealed that both capsular production and viscosity were significantly higher in NTUH-K2044 than in hv-CRKP strains (Figure 6(A,B)). This suggests that the additional capsule-regulatory genes carried by hv-CRKP may not suffice to compensate for the observed disparity in capsule phenotypes between these strains and NTUH-K2044, which likely constitutes a major contributor to the latter’s heightened immune evasion. In addition, siderophore production was evaluated qualitatively using CAS agar plates and quantitatively via CAS liquid assays (Figure 6(C)). On CAS plates, NTUH-K2044 produced the largest halo, approximately 27 mm in diameter, followed by strain D341 with a halo of 23 mm. The remaining hv-CRKP strains produced halos ranging from 20 to 21 mm, whereas HS11286 yielded no detectable halo. Quantitative assessment further demonstrated that siderophore production in NTUH-K2044 was significantly greater than that in all hv-CRKP strains and HS11286. These findings imply that the presence of additional iro and iuc gene clusters in hv-CRKP does not fully reconcile the differential siderophore production between these isolates and NTUH-K2044. Notably, D341, which exhibited the second-highest siderophore output among hv-CRKP strains, also demonstrated superior intestinal colonization in mice compared to other hv-CRKP isolates, suggesting a potential association between siderophore production and colonization efficacy.

(A) Capsule viscosity assay. (B) Uronic acid quantification assay. (C) CAS assays for siderophore analysis. Upper panels: Representative images of CAS agar plates. Lower left: Quantitative analysis of orange halo diameter (mm) from the CAS qualitative assay. Lower right: Quantitative determination of siderophore production via CAS assay solution.

**Figure 7.**
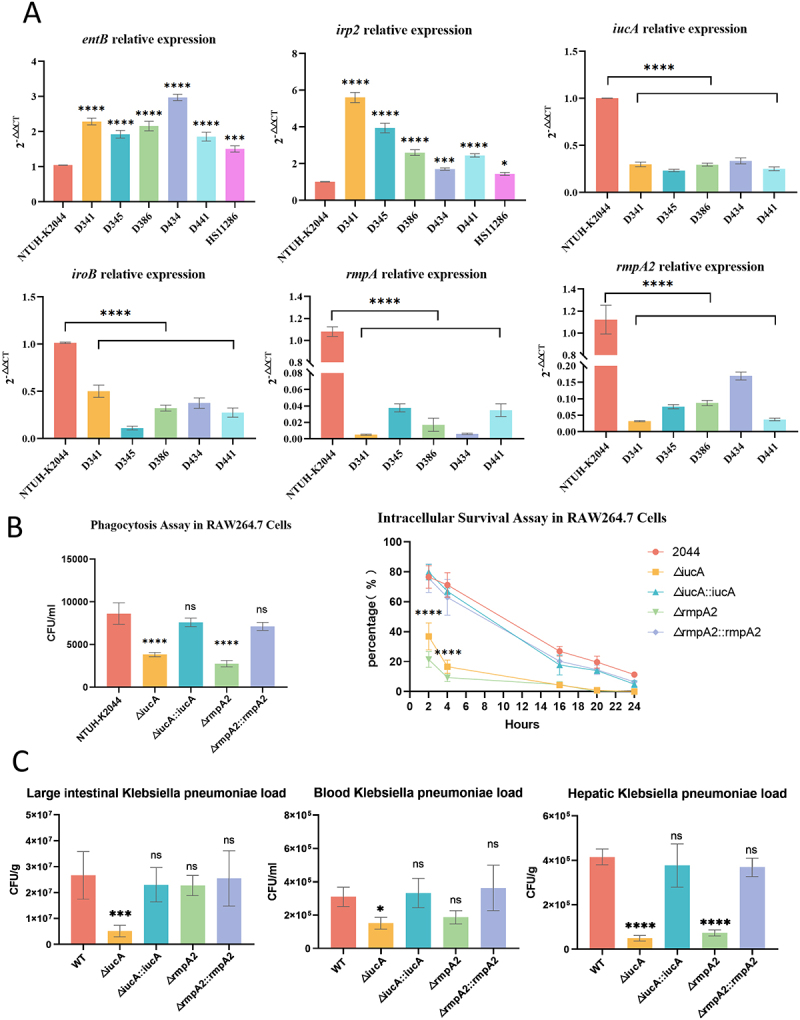
Virulence gene expression, macrophage interaction, and in vivo colonization of *Klebsiella pneumoniae.*

To further explore whether the phenotypic differences in capsule properties and siderophore output were accompanied by transcriptional changes, we compared the expression of representative siderophore-related genes and capsule regulators across NTUH-K2044, hv-CRKP isolates, and HS11286 by RT-qPCR (Figure 7(A)). While hv-CRKP isolates generally exhibited significantly elevated expression of *entB* and *irp2*, the expression of the aerobactin biosynthesis gene *iucA* and the salmochelin-associated gene *iroB* was markedly lower than that in NTUH-K2044. In parallel, the capsule regulatory genes *rmpA* and *rmpA2* were expressed at substantially higher levels in NTUH-K2044 than in hv-CRKP. These transcriptional patterns are consistent with the observed strain-level phenotypes, supporting that differential regulation of capsule-associated pathways and siderophore systems may contribute to the enhanced immune resistance of NTUH-K2044.

(A) RT-qPCR analysis of siderophore-related genes (*entB, irp2, iucA, iroB*) and capsule regulatory genes (*rmpA, rmpA2*) in the indicated K. pneumoniae strains. Relative expression levels are presented as 2^-ΔΔCt values normalized to the reference strain NUTH-K2044.(B) Macrophage assays in RAW264.7 cells using NUTH-K2044, the isogenic mutants (*ΔiucA, ΔrmpA2*) and their complemented strains (*ΔiucA:iucA, ΔrmpA2:rmpA2*). Left: phagocytosis assay quantified by recovered intracellular bacteria (CFU/ml). Right: intracellular survival kinetics presented as survival percentage over time.

(C) Bacterial loads in the large intestine, blood, and liver of infected mice, comparing NTUH‑K2044 wild type, Δ*iucA*and Δ*rmpA2*mutants, and their respective complemented strains (Δ*iucA:iucA*, Δ*rmpA2:rmpA2*).

To causally test the contributions of aerobactin biosynthesis and capsule regulation, we generated isogenic mutants Δ*iucA* and Δ*rmpA2* in the NTUH-K2044 background, along with their corresponding complemented strains. Siderophore production and hypermucoviscosity assays revealed that the Δ*iucA* knockout exhibited a marked reduction in siderophore production, while the Δ*rmpA2* knockout showed a moderate decrease in hypermucoviscosity. Complementation restored both phenotypes (Figure S5). In RAW264.7 macrophages, the intracellular survival assay further demonstrated that deletion of iucA or *rmpA2* significantly reduced intracellular persistence over time, whereas complementation largely restored intracellular survival toward the parental level (Figure 7(B)). In addition, serum resistance was examined for the parental strain, *ΔiucA*, *ΔrmpA2*, and the complemented strains; no significant differences were observed among these strains under our assay conditions (Figure S6). Together, these genetic deletion-and-rescue experiments provide direct support that aerobactin production (*iucA*) and capsule regulation (*rmpA2*) contribute to macrophage-associated fitness, strengthening the mechanistic link between these virulence factors and the immune resistance phenotype observed in hvKp.

We further validated our in vitro findings by assessing in vivo virulence. As shown in Figure 7(C), deletion of *iucA* resulted in significant reductions in bacterial loads in the large intestine, blood, and liver compared to the wild-type strain; the Δ*rmpA2* mutant also showed a significant decrease in the liver, while its loads in the large intestine and blood did not differ significantly from the wild type. Complementation of either gene fully restored bacterial colonization and dissemination to wild-type levels in these tissues. Further analysis of additional tissues and feces (Figure S7) revealed that the Δ*iucA* mutant had significantly impaired colonization in the small intestine, feces, spleen, and lung, indicating that aerobactin plays a critical role in both initial intestinal colonization and systemic dissemination. In contrast, the Δ*rmpA2* mutant exhibited significant reductions in the small intestine, feces, and spleen, whereas its loads in the lung were comparable to the wild type, suggesting that RmpA2 contributes to colonization of specific niches and deep organ infection, but is not strictly required for pulmonary dissemination.

Collectively, these experiments confirm that aerobactin production (iucA) and capsule regulation (rmpA2) jointly contribute to bacterial macrophage-associated fitness and in vivo colonization and dissemination, further strengthening the mechanistic link between these virulence factors and the immune resistance phenotype of hvKp.

## Discussion

The emergence of hv-CRKP represents a significant public health concern. In this study, analysis of 847 clinical isolates from four Chinese hospitals identified 157 (18.5%) strains co-harboring key virulence genes (*rmpA/rmpA2* and *iucA/iroB*) and carbapenem resistance genes, with ST11-K64 being the predominant type within our cohort and sampling period. This prevalence underscores the considerable clinical challenge posed by these convergent strains. Subsequent experimental investigations revealed that the five representative ST11-K64 hv-CRKP isolates exhibited attenuated virulence compared to the classical hypervirulent reference strain NTUH-K2044 (ST23-K1) in the murine models tested in this study. Specifically, in a murine model simulating the natural entero-hematogenous-hepatic pathway, these hv-CRKP strains were severely impaired in intestinal colonization, systemic dissemination, and hepatic survival, failing to induce pyogenic liver abscesses.

These findings align with Kochan et al. [10], who reported low virulence in US convergent isolates (mainly ST15/ST16) in a pneumonia model. However, our study focuses on the Asian-prevalent ST11-K64 clone [23] and employs an intragastric model that directly evaluates intestinal translocation and liver abscess formation – a hallmark of classical hypervirulence [24]. This approach revealed specific deficits in the liver infection pathway, complemented by cellular and phenotypic assays elucidating the underlying mechanisms.

Bacterial loads of NTUH-K2044 in both feces and the large intestine were higher than those of hv-CRKP. Although NTUH-K2044 exhibited weaker adhesion to and invasion of Caco-2 cells compared to hv-CRKP, it demonstrated superior resistance to intestinal immune clearance. Phagocytosis and intracellular survival assays using RAW264.7 macrophages indicated that NTUH-K2044 was less readily phagocytosed and exhibited higher post-phagocytosis survival rates than hv-CRKP. This enhanced immune evasion might correlate with capsular characteristics: hv-CRKP produced significantly less capsule with lower viscosity than NTUH-K2044, and a thick capsule is crucial for resisting macrophage capture [25]. Furthermore, nutritional immunity, particularly iron acquisition, plays a vital role in gut colonization [26]. NTUH-K2044 produced greater amounts of siderophores, potentially providing a competitive advantage in the iron-limited gut environment [2]. Consistent with these phenotypes, RT-qPCR revealed distinct expression patterns of capsule regulators (*rmpA/rmpA2*) and siderophore-associated genes (including *iucA/iroB*) between NTUH-K2044 and hv-CRKP isolates, supporting divergent regulation of these virulence genes across genetic backgrounds.

In the bloodstream, only NTUH-K2044 could effectively resist complement-mediated serum killing and sustain proliferation. Its thick capsule can act as a physical barrier impeding the deposition of C3b and the membrane attack complex, thereby inhibiting complement-mediated bacterial lysis [27,28]. Interestingly, deletion of *iucA* or *rmpA2* in the NTUH-K2044 background did not significantly affect serum survival under our assay conditions (Figure S6). In contrast, we previously observed that deletion of the capsule biosynthesis gene wcaJ markedly reduced serum resistance in NTUH-K2044 [19], suggesting that serum survival is more sensitive to major disruptions in capsule biosynthesis than to the regulatory modulation achieved by rmpA2 deletion. Of note, although most ST11-KL64 isolates did not reach the bloodstream, two isolates (D341 and D345) from the same clade did cause bacteremia in the oral entero‑hepatic model, suggesting some intra‑lineage variability in the capacity for extraintestinal dissemination.

In the liver, only NTUH-K2044 established a persistent presence. Its advantage was not attributable to enhanced adhesion or invasion of HepG2 cells but rather to a significantly stronger ability to resist phagocytosis by ImKCs. Kupffer cells are a critical defense mechanism in the liver [29,30], and the resistance to their capture and killing varies with capsule type [31,32]. After phagocytosis, NTUH-K2044 maintained a growth trend within the first 4 h, whereas other strains declined rapidly. Over longer periods, the decrease in intracellular bacterial loads across all strains may be related to macrophage pyroptosis following infection. Pyroptosis leads to membrane rupture and bacterial release, potentially exacerbating tissue damage [33,34]. The released hvKp, demonstrating enhanced resistance to neutrophil attacks and immune bactericidal factors [35–37], can also directly inflict damage on hepatocytes, inducing apoptosis and necrosis [38], collectively contributing to liver abscess formation.

The attenuated entero-hepatic pathogenicity of ST11-K64 observed in our model appears to contrast with clinical reports of severe extraintestinal infections caused by ST11 hv-CRKP [6]. This discrepancy underscores the context-dependent nature of hypervirulence and can be reconciled by considering the infection route. The virulence plasmid may confer a decisive advantage when the intestinal barrier is bypassed, such as in respiratory tract infections or in immunocompromised hosts, explaining its severe disease potential in those settings. This context-specificity also highlights the limitations of our study, which primarily relies on an entero-hepatic model for defining hypervirulence. A multi-model assessment, including respiratory and immunocompromised host models, is therefore needed to fully capture the pathogenic potential of these convergent strains.

To explain the mechanistic basis for their reduced intestinal translocation and liver tropism, we propose several non-mutually exclusive hypotheses. First, our sequence comparisons revealed no SNPs in *iucA*, *iroB*, or *rmpA2* between ST11-KL64 and NTUH-K2044 (Figure S8 and Figure S9), and the potential *rmpA* truncation observed by short-read sequencing requires long-read validation. Given that coding-sequence mutations are unlikely to explain the reduced expression, we suspect that differences in virulence plasmid structure or copy number may be involved [39]. Elucidating the individual and combined contributions of these plasmid-, chromosome-, and regulation-based mechanisms represents a major focus of future investigations. Second, the unique chromosomal background of ST23 strains may harbor, beyond the K1 capsule gene cluster [28], other allelic variants that confer critical fitness advantages for gut colonization and hepatic invasion. Third, considering the established role of non-coding small RNAs in modulating capsular biosynthesis [40], differences in the expression or function of these regulatory molecules between ST23 and ST11 genetic backgrounds could substantially alter virulence factor production. Elucidating the individual and combined contributions of these plasmid-, chromosome-, and regulation-based mechanisms represents a major focus of future investigations.

Our epidemiological estimates and clonal distributions are based on four hospitals within a single sampling window, and broader geographic and longitudinal surveillances are needed to assess representativeness and temporal stability. Most ST11-K64 hv-CRKP isolates were recovered from respiratory specimens, whereas our primary *in vivo* work used an oral entero-hepatic model; thus, the findings should be interpreted as route-specific phenotypes under the conditions tested. Capsule (K-type) and virulence-marker assignments were inferred from short-read whole-genome sequencing data, and we did not perform isolate-by-isolate functional confirmation across the entire collection; targeted validation strategies will therefore be required to better define the population-level frequency of functional hypervirulence determinants and high-confidence K-type assignments. Finally, only a limited number of ST11-K64 isolates were tested in vivo, and expanded sampling, particularly including ST11 hv-CRKP from confirmed invasive infections and from different phylogenetic clades, together with complementary, route-matched models and long-read sequencing, will be important to strengthen lineage-associated conclusions and resolve plasmid structures and validate potential gene truncations.

In conclusion, this study demonstrates that the predominant ST11-K64 hv-CRKP strains in our cohort are attenuated in their ability to cause invasive liver abscesses via the intestinal route under the conditions tested, primarily due to impairments in immune evasion, potentially linked to deficits in capsule production and siderophore activity. This suggests that the convergence of resistance and virulence genes does not automatically confer the full invasive potential of classical hvKp. However, the retained capacity of these strains to cause severe extraintestinal infections necessitates continued vigilance. Future research should prioritize elucidating the genetic basis of virulence attenuation and employ multi-model systems to comprehensively evaluate the pathogenic potential of these evolving threats.

## Supplementary Material

Author Checklist .pdf

Cleansupplement(2).docx

## Data access

The bam files of whole-genome resequencing were deposited at the NCBI Sequence Read Archive (SRA) with the BioProject accession number PRJNA1297889, PRJNA1332357, PRJNA1332349 and PRJNA1332406. All original data are provided and publicly accessible via Figshare. (DOIs: 10.6084/m9.figshare.30394162)
