# Supplementary material for: Virulence attenuation in ST11-K64 Klebsiella pneumoniae explains its divergent clinical manifestation from ST23-K1
Source: Virulence. 2026 Jun 26;17(1):2690744. doi: 10.1080/21505594.2026.2690744 (PMC13313197; doi:10.1080/21505594.2026.2690744)
Supplement: Cleansupplement(2).docx [file KVIR_A_2690744_SM6294.docx]

**Supplementary Material**

TABLE S1

Typing and MIC of hypervirulent carbapenem-resistant isolates and reference strains

| isolates | NTUH-K2044 | 11286 | D341 | D345 | D386 | D434 | D441 |
| --- | --- | --- | --- | --- | --- | --- | --- |
| Typing |  |  |  |  |  |  |  |
| Capsule antigen serotype | K1 | KL47 | K64 | K64 | K64 | K64 | K64 |
| MLST | ST23 | ST11 | ST11 | ST11 | ST11 | ST11 | ST11 |
| MIC (mg/L) |  |  |  |  |  |  |  |
| Ampicillin | 16 | >32 | >32 | >32 | >32 | >32 | >32 |
| Cefoxitin | ≤8 | 16 | >32 | >32 | >32 | >32 | >32 |
| Cefazolin | ≤2 | >32 | >32 | >32 | >32 | >32 | >32 |
| Cefuroxime | ≤4 | >16 | >16 | 16 | >16 | >16 | >16 |
| Ceftazidime | ≤1 | 16 | >16 | >16 | >16 | >16 | >16 |
| Cefotaxime | ≤0.12 | >4 | >4 | >4 | >4 | >4 | >4 |
| Cefepime | ≤0.5 | 16 | >16 | >16 | >16 | >16 | >16 |
| Ceftazidime/Avibactam | ≤0.5/4 | ≤0.5/4 | 1/4 | ≤0.5/4 | ≤0.5/4 | 1/4 | ≤0.5/4 |
| Cefoperazone/Sulbactam | ≤16/8 | >64/32 | >64/32 | >64/32 | >64/32 | >64/32 | >64/32 |
| Piperacillin/Tazobactam | ≤8/4 | >64/4 | >64/4 | >64/4 | >64/4 | >64/4 | >64/4 |
| Ampicillin/Sulbactam | ≤8/4 | >32/16 | >32/16 | >32/16 | >32/16 | >32/16 | >32/16 |
| Amoxicillin/Clavulanate Potassium | ≤8/4 | >32/16 | >32/16 | >32/16 | >32/16 | >32/16 | >32/16 |
| Meropenem | ≤0.06 | 16 | >16 | >16 | >16 | >16 | >16 |
| Imipenem | ≤0.25 | 16 | >16 | >16 | >16 | >16 | >16 |
| Ertapenem | ≤0.015 | >2 | >2 | >2 | >2 | >2 | >2 |
| Moxifloxacin | ≤0.25 | >2 | >2 | >2 | >2 | >2 | >2 |
| Levofloxacin | ≤0.12 | 2 | >8 | >8 | >8 | >8 | >8 |
| Tigecycline | 0.5 | 0.5 | 1 | 0.5 | 0.5 | 0.5 | 0.5 |
| Tetracycline | ≤2 | 16 | ≤2 | ≤2 | ≤2 | ≤2 | ≤2 |
| Gentamicin | ≤1 | >16 | >16 | >16 | >16 | >16 | ≤1 |
| Amikacin | ≤16 | >64 | >64 | >64 | >64 | >64 | ≤16 |
| Aztreonam | ≤4 | >16 | >16 | >16 | >16 | >16 | >16 |
| Nitrofurantoin | ≥64 | >64 | >64 | >64 | >64 | >64 | >64 |
| Compound Sulfamethoxazole | ≤0.5/0.5 | >4/76 | >4/76 | >4/76 | >4/76 | >4/76 | >4/76 |
| Polymyxin | 0.032 | 0.047 | 0.047 | 0.064 | 0.032 | 0.032 | 0.032 |


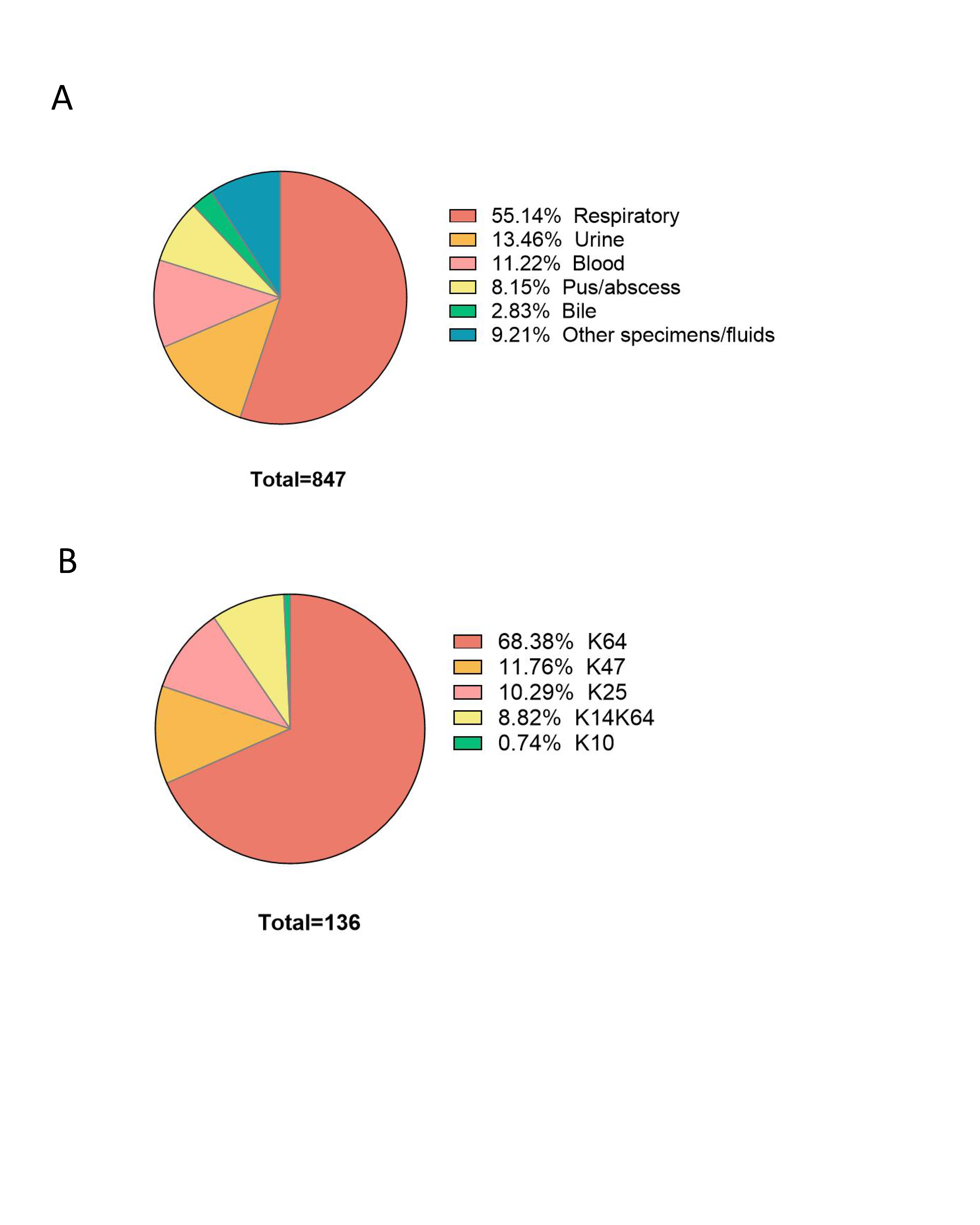


Figure S1. Specimen sources of the clinical isolate collection and capsule serotype distribution of ST11 hv-CRKP isolates.

(A) Distribution of specimen sources for the 846 clinical Klebsiella pneumoniae isolates included in this study.

(B) Capsule serotype (K type) distribution among ST11 isolates within the hv-CRKP collection.


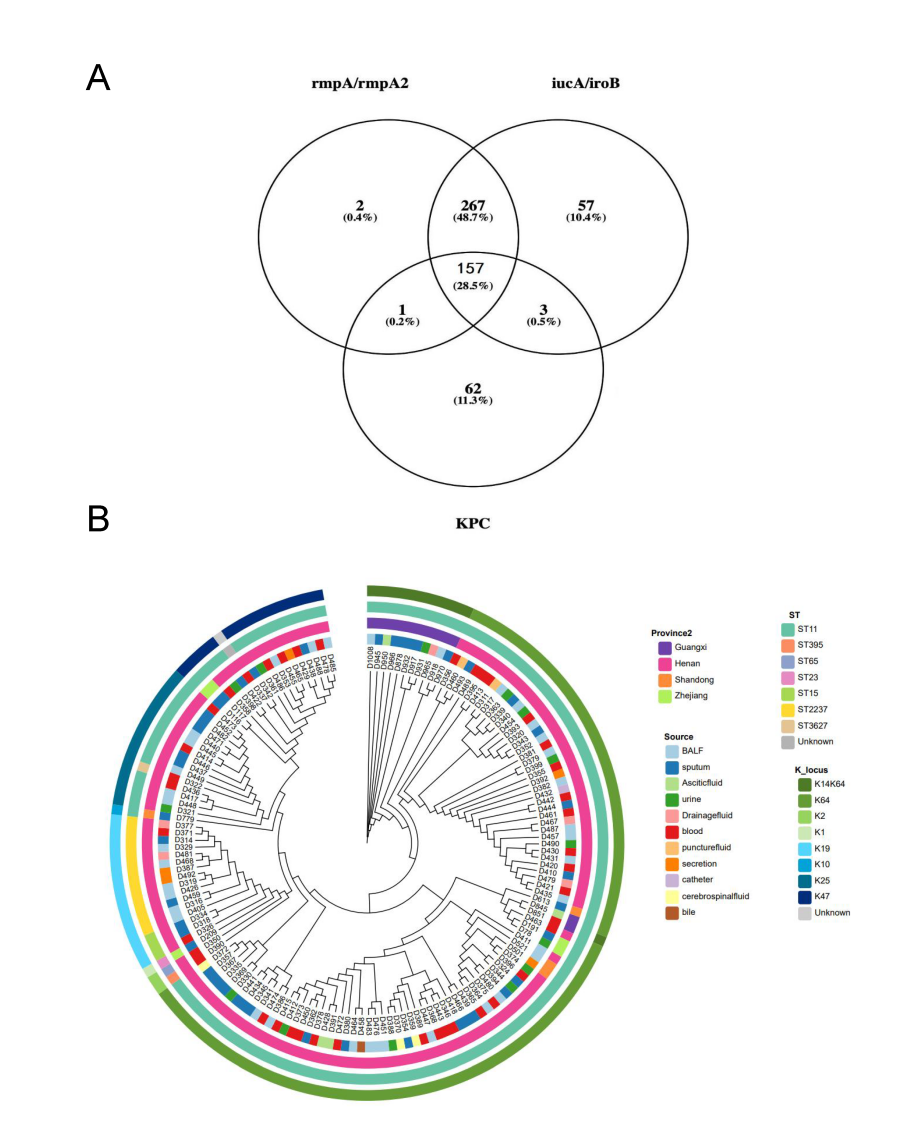


Figure S2. Characterization of 157 hv-CRKP Isolates

1. Venn diagram illustrating the co - occurrence patterns of hypervirulence - associated and the carbapenem resistance gene KPC among *Klebsiella pneumoniae* isolates.genes. (B) Circular hierarchical plot showing the evolutionary tree, provincial origin, specimen source, ST, and K types of 157 *Klebsiella pneumoniae* isolates with concurrent hypervirulence and KPC carriage.


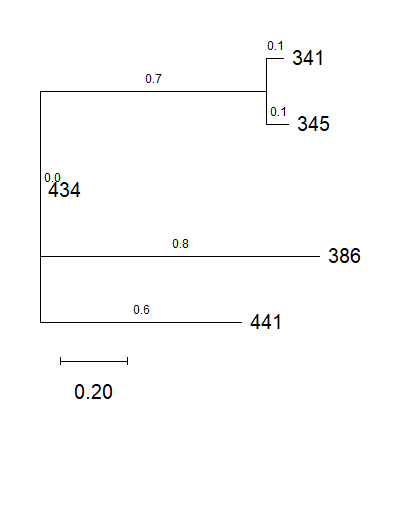


Figure S3. Core-genome SNP phylogeny of hv-CRKP isolates chosen for future experiments.

Phylogenetic tree constructed from core-genome SNPs of hv-CRKP isolates (D341, D345, D386, D434, and D441) using kSNP4. The tree was visualized in MEGA. Numbers shown on branches represent branch lengths (genetic distances), expressed as estimated substitutions per site.

**
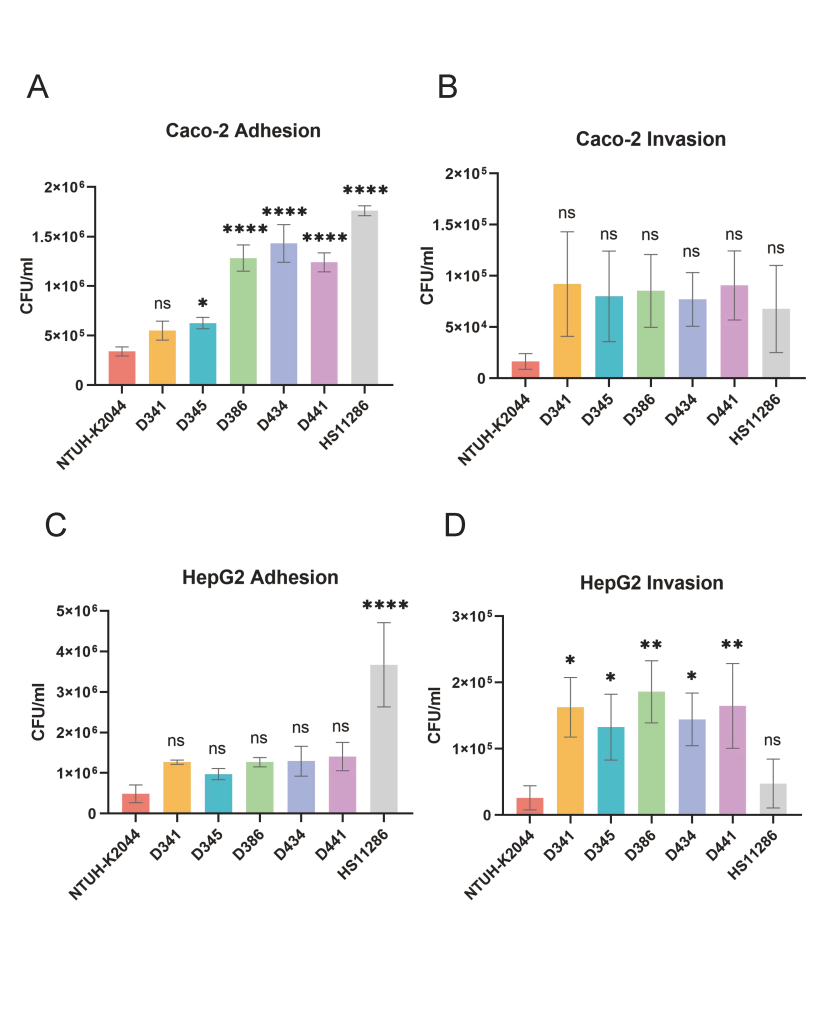
**

Figure S4. Adhesion and invasion capacities of different *Klebsiella pneumoniae* strains in Caco-2 and HepG2 cell lines.

1. Adhesion of K. pneumoniae strains to Caco-2 cells. (B) Invasion of K. pneumoniae strains into Caco-2 cells. (C) Adhesion of K. pneumoniae strains to HepG2 cells. (D) Invasion of K. pneumoniae strains into HepG2 cells.


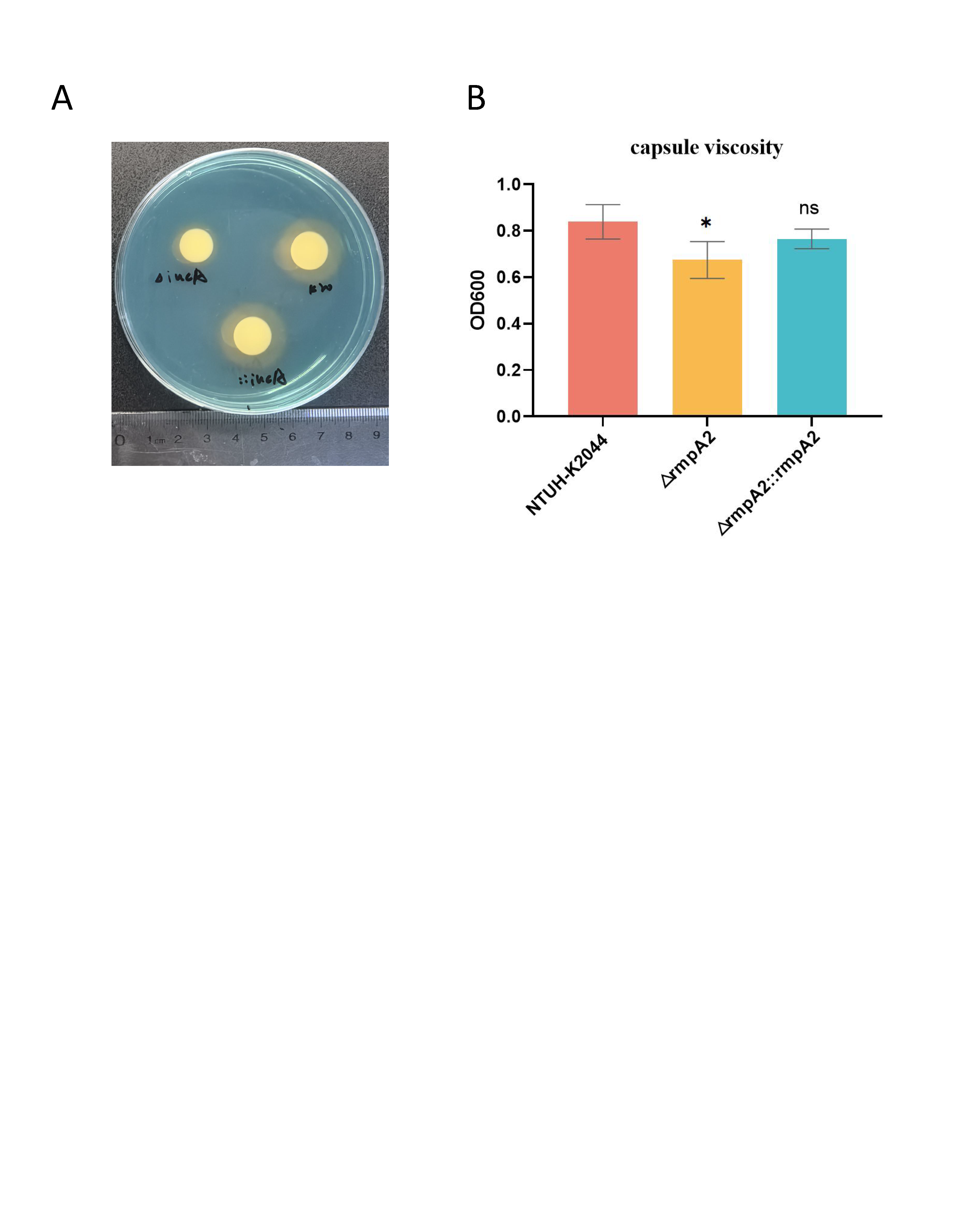


Figure S5. Phenotypic validation of siderophore production and capsule viscosity in NTUH-K2044 and its isogenic mutants.

(A) CAS agar plate showing siderophore production by NTUH-K2044, Δ*iucA*, and the complemented strain Δ*iucA::iucA* (halo formation indicates siderophore activity). (B) Capsule viscosity assay of NTUH-K2044, Δ*rmpA2*, and the complemented strain Δ*rmpA2::rmpA2*, quantified by OD600. Statistical significance is indicated as P < 0.05 (*) and not significant (ns).


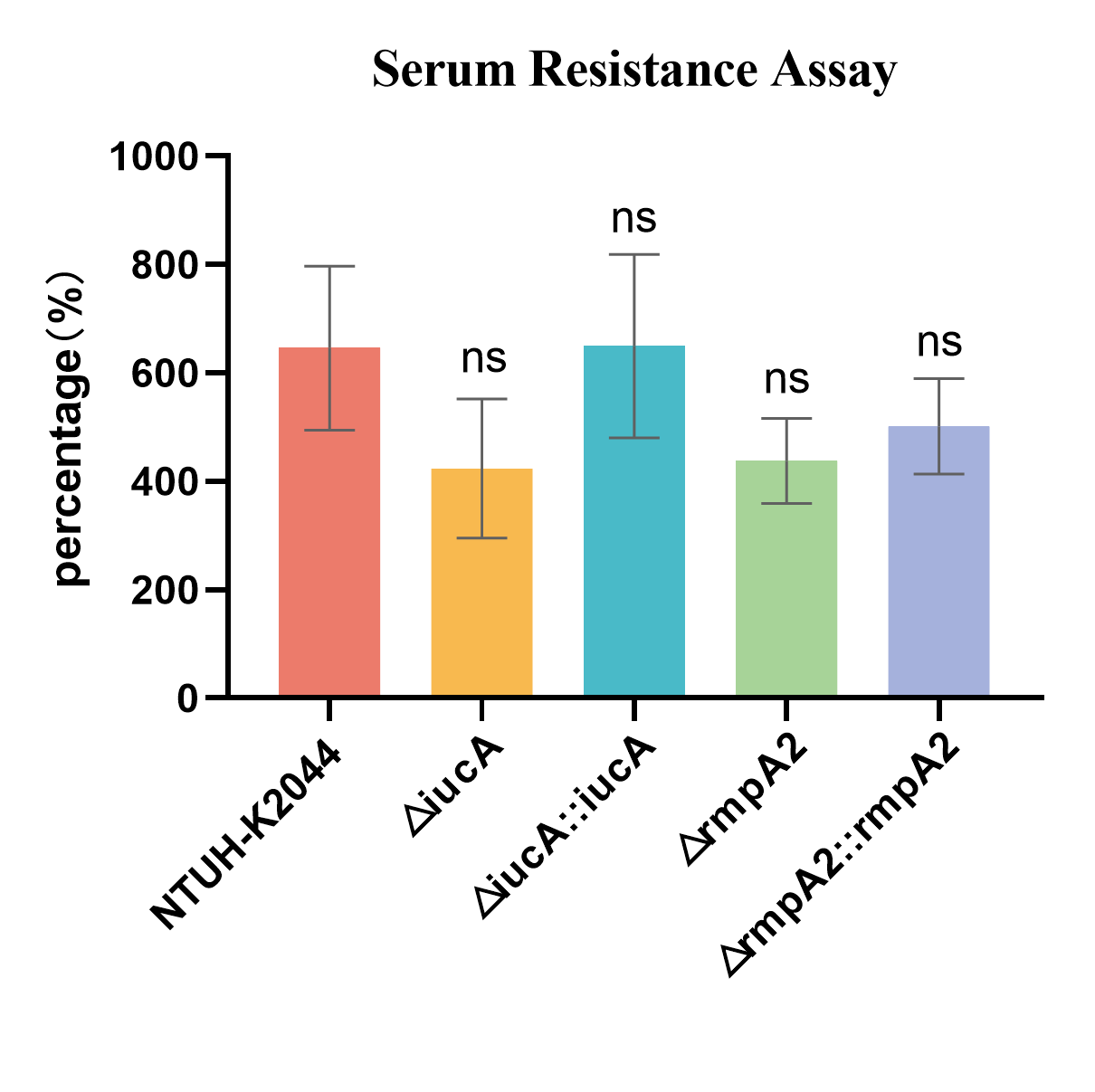


Figure S6. Serum resistance of NTUH-K2044 and its isogenic mutants.

Serum resistance assay of NTUH-K2044, the isogenic mutants (Δ*iucA*, Δ*rmpA2*) and the corresponding complemented strains (Δ*iucA::iucA*, Δ*rmpA2::rmpA2*). Bacterial survival is presented as percentage (%) after serum exposure. Bars indicate mean ± SD from independent experiments. ns indicates no statistically significant difference compared with NTUH-K2044 under the tested conditions.


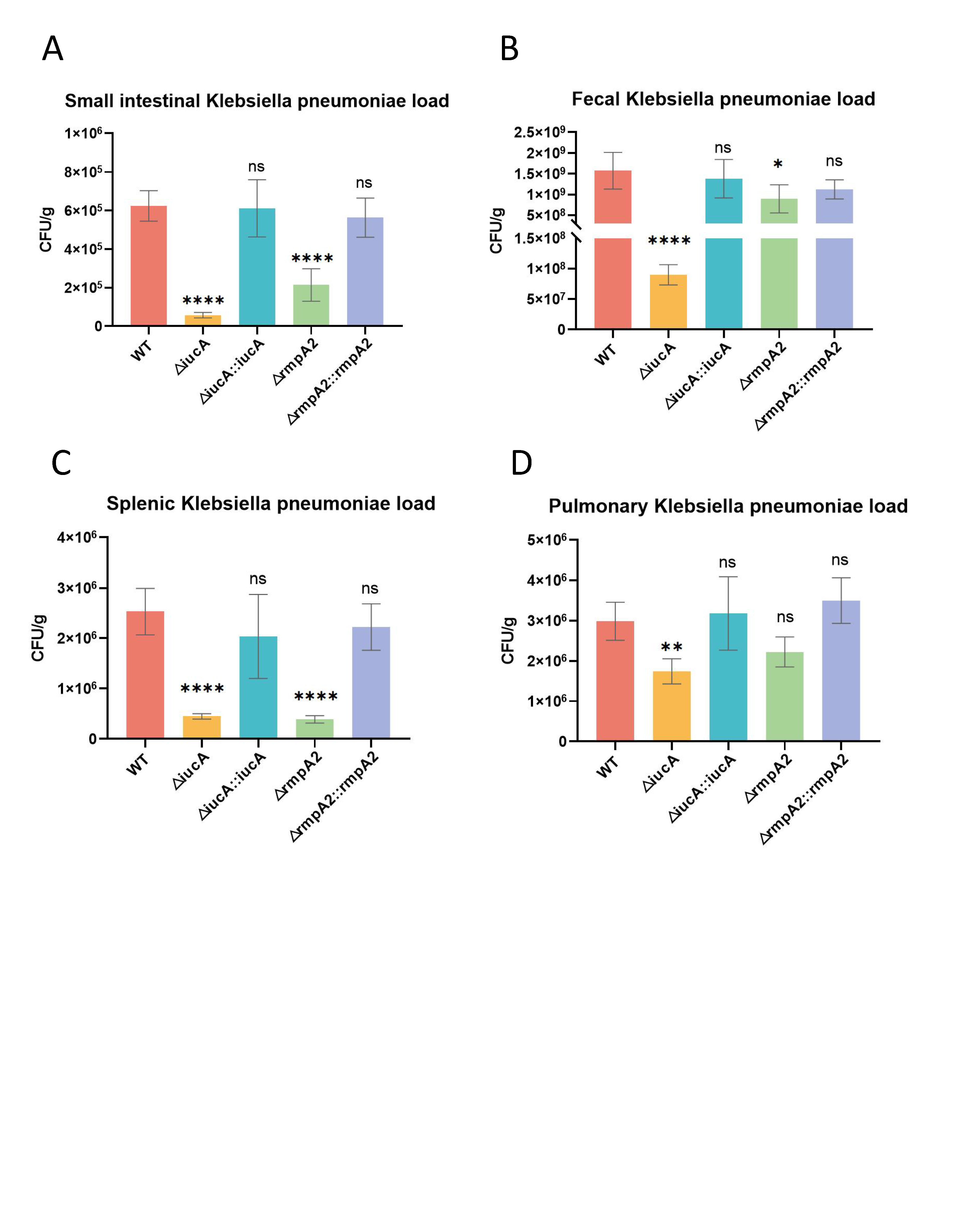


Figure S7. Bacterial Colonization and Dissemination of NTUH-K2044 and its isogenic mutants.

1. Small intestinal, (B) fecal, (C) splenic, and (D) pulmonary bacterial loads in infected mice, comparing NTUH‑K2044 wild type, Δ*iucA* and Δ*rmpA2* mutants, and their respective complemented strains (Δ*iucA::iucA*, Δ*rmpA2::rmpA2*).


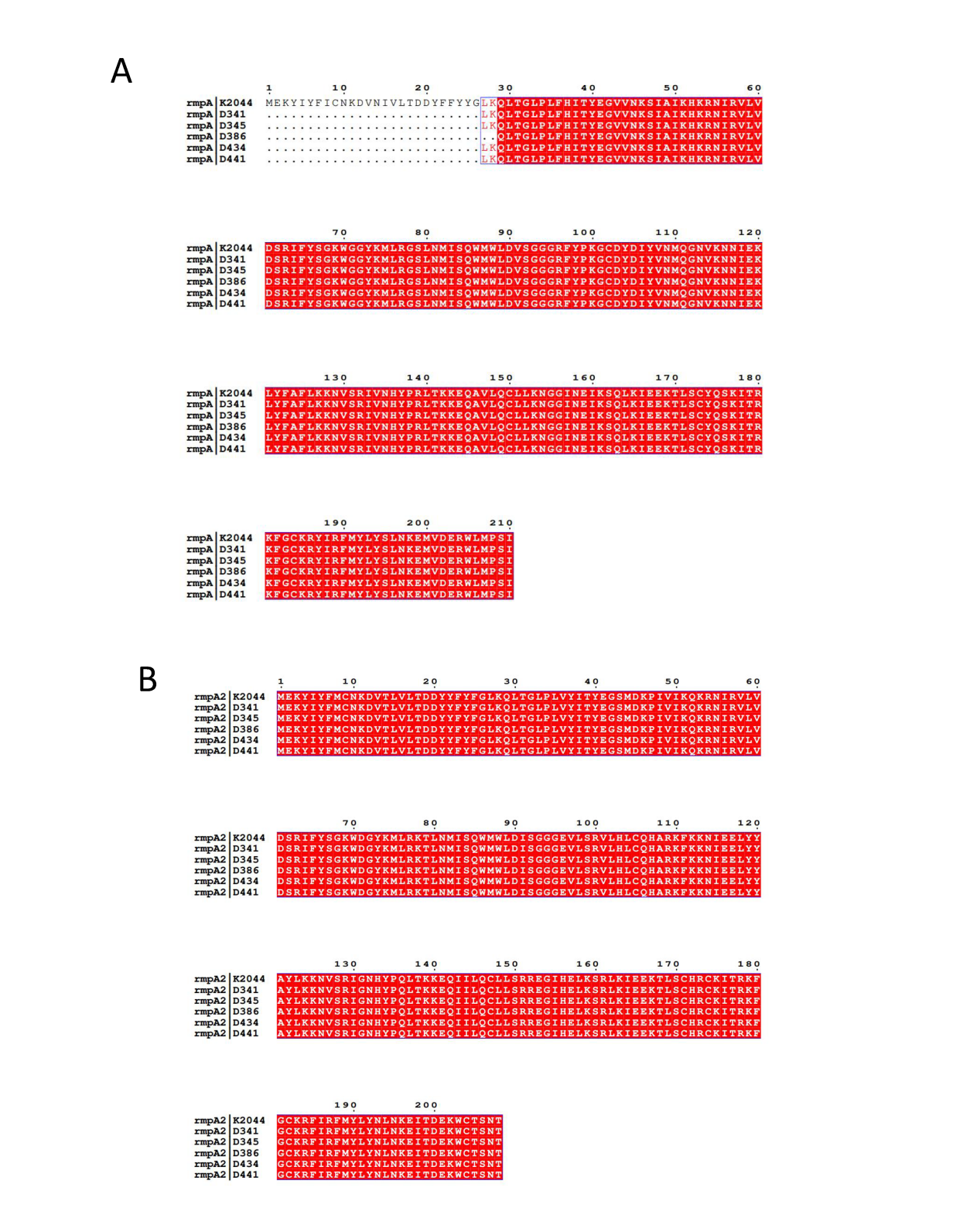


Figure S8.Amino acid sequence alignment of RmpA and RmpA2 proteins

1. Alignment of RmpA amino acid sequences from the hypervirulent reference strain Klebsiella pneumoniae NTUH-K2044 and five clinical ST11-K64 CR-hvKp isolates (D341, D345, D386, D34, D441). (B) Alignment of RmpA2 amino acid sequences from the same panel of strains. Identical amino acid residues are shaded in red. No amino acid variations were detected in RmpA2 across all tested strains. Sequence alignments were generated using Clustal Omega and visualized with ESPript 3.2.


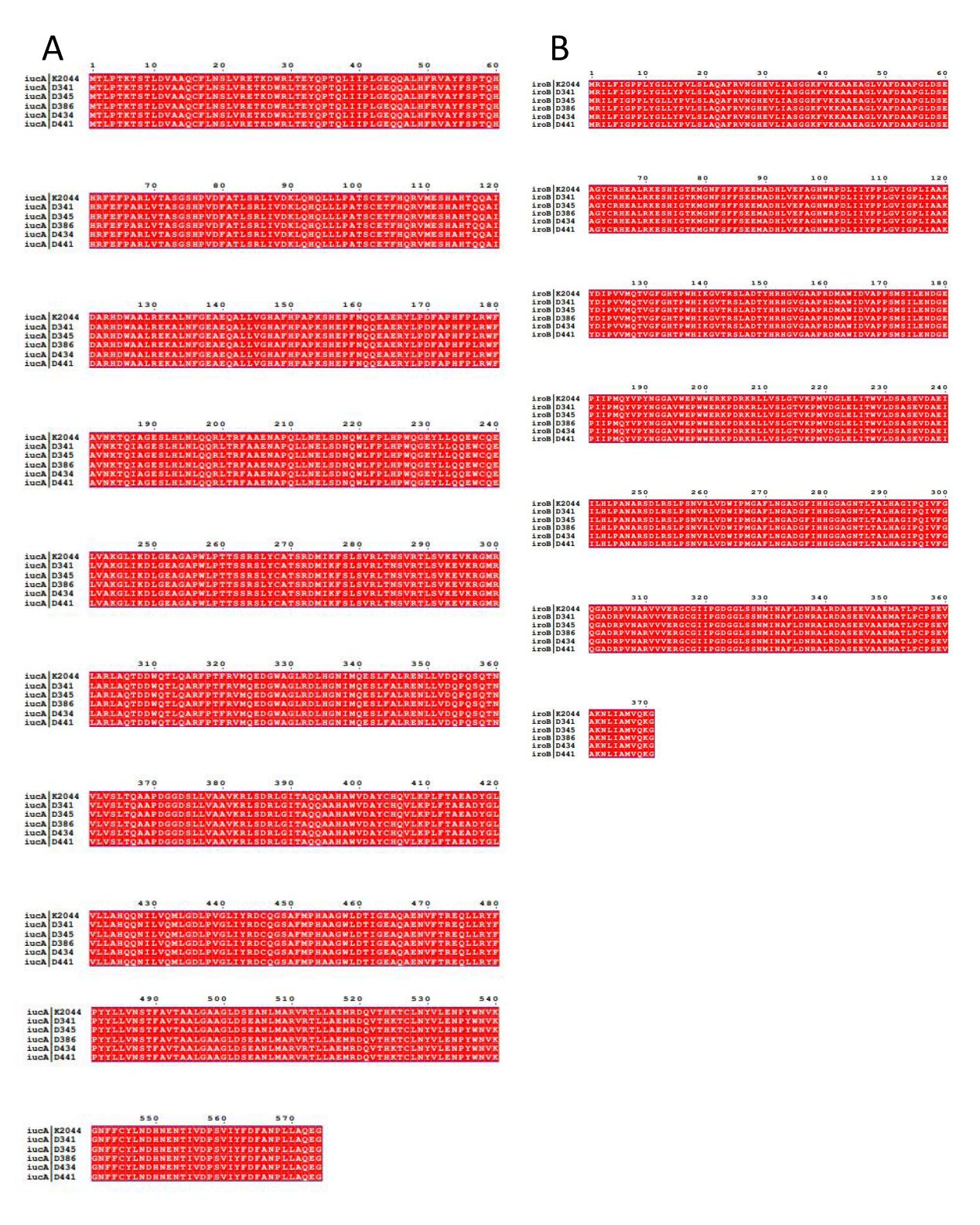


Figure S9.Amino acid sequence alignment of IucA and IroB proteins

1. Alignment of IucA amino acid sequences from the hypervirulent reference strain Klebsiella pneumoniae NTUH-K2044 and five clinical ST11-K64 CR-hvKp isolates (D341, D345, D386, D34, D441). (B) Alignment of IroB amino acid sequences from the same panel of strains. Identical amino acid residues are shaded in red. No amino acid variations were detected in IucA and IroB across all tested strains. Sequence alignments were generated using Clustal Omega and visualized with ESPript 3.2.
